# Acellular dermal matrix in urethral reconstruction

**DOI:** 10.3389/fped.2024.1342906

**Published:** 2024-02-09

**Authors:** Jiang Aodi, Lian Ying, Sun Chengyang, Zhai Hongfeng

**Affiliations:** Department of Plastic and Aesthetic Surgery, People’s Hospital of Henan University, People’s Hospital of Zhengzhou University, Henan Provincial People’s Hospital, Zhengzhou, China

**Keywords:** acellular dermal matrix, tissue engineering, urethral strictures, hypospadias, regeneration

## Abstract

The management of severe urethral stricture has always posed a formidable challenge. Traditional approaches such as skin flaps, mucosal grafts, and urethroplasty may not be suitable for lengthy and intricate strictures. In the past two decades, tissue engineering solutions utilizing acellular dermal matrix have emerged as potential alternatives. Acellular dermal matrix (ADM) is a non-immunogenic biological collagen scaffold that has demonstrated its ability to induce layer-by-layer tissue regeneration. The application of ADM in urethral reconstruction through tissue engineering has become a practical endeavor. This article provides an overview of the preparation, characteristics, advantages, and disadvantages of ADM along with its utilization in urethral reconstruction via tissue engineering.

## 1 Introduction

The urethra is a tubular structure that connects the urinary bladder to the external environment. It primarily consists of two distinct cell types: epithelial cells and smooth muscle cells. In comparison to the female urethra, the male urethra is slender and can be anatomically divided into penile (cavernous), bulbous, membranous, and prostatic segments (1). There are multiple etiologies for urethral injury, including iatrogenic causes, infections, trauma, tumors, and congenital hypospadias (2, 3). Hypospadias is a common congenital malformation that occurs in approximately ∼1/150–1/300 live births (3). Following urethral injury, the organization and distribution of fibroblasts in normal tissues become disrupted, leading to the development of urethral strictures (4). Patients may experience abnormal urination, pain, urinary tract infections, and potential impairment of kidney function or overall quality of life. Anastomotic urethroplasty (AR), urethral dilation (UD), or direct vision internal urethrotomy (DVIU) are commonly employed treatment options for managing urethral strictures. Steenkamp et al. (5) described changes in sexual function and reproductive sensitivity following AR repair for bulbous urethral stricture; temporary decline in erectile and ejaculatory function can occur as a result of AR intervention. Moreover, both UD and DVIU exhibit reduced efficacy with increasing length of the stricture. Historically oral mucosal grafts considered as “gold standard” materials for repairing urethral strictures or defects due to their favorable therapeutic outcomes; autologous replacement tissues such as penile or scrotal skin grafts bladder mucosa grafts, and oral mucosal grafts often give rise to various complications at the donor site (6).

In the past two decades, with the advancement of tissue engineering, various strategies have been proposed to address the challenge of limited sources of autogenous tissue for urethral reconstruction (7, 8). Tissue engineering is a scientific discipline that applies principles from cell biology and engineering to develop active biological substitutes capable of repairing damaged tissues and enhancing their functionality. Its fundamental principle involves creating a three-dimensional complex composed of cells and biological materials (9). Numerous biodegradable materials are currently under investigation, including acellular matrix, polylactic acid (PLA), polyglycolic acid (PGA), as well as PLA-PGA copolymers (10–12). Hu et al. (13) described the biocompatibility and application of polylactic acid-glycolic acid copolymer (PLGA) and PLGA-collagen scaffolds in canine urethral reconstruction. Although urothelial cells implanted on both scaffolds exhibited satisfactory growth, resulting in multiple layers formation within proximal and distal segments of the reconstructed urethra, post-surgical complications such as strictures and urethral discontinuities were observed. It is evident that the performance of PLGA and PLGA-collagen scaffolds was suboptimal. The acidic degradation products commonly associated with synthetic polymeric materials, such as PLGA, may impede proper cellular growth in the surrounding environment (14). However, acellular matrices closely resemble the native extracellular matrix, making acellular dermal matrix (ADM) an innovative treatment approach to overcome these challenges in urethral reconstruction. This article presents a comprehensive review on the preparation and characteristics of ADM materials, along with their research advancements in tissue engineering for urethral reconstruction.

## 2 Preparation of ADM

ADM is produced using acellular techniques that involve the enzymatic removal of the epidermis and complete elimination of residual cells from the dermis, resulting in an acellular collagen-elastin biomaterial matrix with minimal antigenicity and exceptional biological and mechanical properties (15). Various physical, chemical, and biological methods are currently employed for preparing ADM (Refer to Table 1).

**Table 1:** Categorization of acellular dermal matrix preparation methods.

Methods	Primary effect
Physical	Cells are eliminated through the combined action of apoptotic cells and cell-matrix adhesion proteins
Chemical	Disrupts cellular membranes and degrades genetic material.
Biological	Reactive cleavage of the arginine or lysine carboxyl side chains in cell adhesion proteins leads to cellular detachment and lysis from the adjacent extracellular matrix

### 2.1 Physical methods

The physical method disrupts the cell membrane, induces cell lysis, and facilitates the transport of cellular debris (16–19). Commonly employed physical methods include repeated cycles of freezing and thawing, ultrasonic vibration, supercritical carbon dioxide, high hydrostatic pressure treatment, mechanical compression, and electroporation. In recent years, numerous studies have attempted to utilize physical techniques in facilitating decellularization and demonstrated its feasibility.

The freeze-thaw method can be optimized by increasing the temperature differential or adjusting the number of cycles, thereby enhancing its efficiency. Multiple freeze-thaw cycles may be employed throughout the decellularization process without compromising matrix protein preservation (20). It is important to note that complete removal of nuclear material should not be reported, as the formation of ice crystals during freezing and thawing disrupts cell membranes in tissues or organs (21). While this process effectively preserves biochemical composition and mechanical properties, insufficient removal of genetic material may lead to potential immunological rejection. To disrupt cell membranes, high hydrostatic pressure exceeding 600 MPa is applied; however, it should be acknowledged that such high pressure can induce protein deformation, as evidenced by observed collagen and elastic fiber deformations in decellularized blood vessels. Consequently, this results in a reduction of approximately 50% in the tensile strength of fibers compared to their original tissue state (22).

Supercritical carbon dioxide exhibits low viscosity and high transport properties, allowing for minimal damage to tissue mechanical properties when passed through at a controlled speed similar to the critical point. Various studies have demonstrated its ability to induce non-deformability in tissue fibers (23). However, its poor solubility for polymers and polar substances is a drawback that can be partially addressed with entraining agents such as ethanol, though this may introduce new impurities (24).

Nonthermal irreversible electroporation, typically achieved through the application of microsecond electrical pulses, disrupts the transmembrane electrical potential and induces micropore formation in the plasma membrane. Ultimately, this leads to cell death by perturbing its steady-state electrical balance while largely preserving the three-dimensional structure of tissues and organs. A novel porcine acellular dermal matrix (PADM) prepared by Xia et al. (25), utilizing laser micropore technology, was demonstrated to be both safe and effective in animal transplantation.

### 2.2 Chemical methods

Chemical decellularization methods commonly employ a variety of detergents, including surfactants, acids, bases, hypertonic and hypotonic solutions, and chelating agents. Surfactants can be classified based on their charge as ionic, nonionic or zwitterionic; all of which have the ability to disrupt cell membranes and degrade DNA. Hogg et al. (26) utilized chemical treatment to remove the epidermis, followed by immersion in a hypotonic buffer solution for cell dissolution and subsequent addition of nuclease buffer to eliminate residual nucleic acid substances. This approach achieved rapid and efficient preparation of ADM. Draguňova et al. (27) also developed an effective method for preparing decellularized matrices using only a few chemicals with minimal procedures. Bera et al. (28) employed a method based on hypotonic/hypertonic saline solution to decellularize goat skin before formulating an ADM bio-ink and 3D bioprinting it. Ultimately, this resulted in an ADM with exceptional mechanical properties and cell adhesion.

### 2.3 Biological methods

Organisms (enzymes) selectively cleave the arginine carboxyl side of cell adhesion proteins, resulting in detachment and lysis of cells from the adjacent matrix. Commonly employed enzymes include trypsin, collagenase, nuclease, thermophilic protease, and dispase (29, 30). Excessive utilization of enzymes may lead to the degradation of natural matrix components, including collagen, elastin, and glycosaminoglycans (31). Following enzymatic decellularization, thorough rinsing of the tissue is essential to eliminate or neutralize any residual enzyme components and cellular debris (32). Therefore, it is recommended to use trypsin for short-term periods to prevent damage to the matrix components. It should be noted that trypsin does not exhibit cytotoxic effects on bioengineered materials which are crucial for *in vitro* cell culture.

As each method possesses its own set of advantages and disadvantages, a combination of methodologies can be employed to explore a more efficacious acellular process and prepare an ideal ADM with enhanced decellularization efficiency. While ionic SDS has demonstrated commendable efficacy in eluting cells and removing cellular components, it also leads to the degradation of the extracellular matrix due to the elution of other substances. Triton X-100, a nonionic eluent, exhibits inefficacy against the eluted cells while causing minimal damage to the extracellular matrix. Although enzymatic hydrolysis can selectively eliminate DNA and other cellular components, its sole effect on elution is suboptimal and may result in certain damages to blood vessels and ultrastructure. Therefore, combining physical, chemical, and enzymatic methods can yield superior outcomes in terms of decellularization.

## 3 Sources of ADM

The sources of ADM can vary, including human, porcine, bovine, ovine or piscine origins (33–37). Human-derived ADMs require tissue screening for infectious pathogens such as HIV, hepatitis, and syphilis. However, the limited availability and high costs significantly restrict their utilization (38). Additionally, the presence of residual heterologous antigens like α-1,3-galactose (α-Gal) may trigger an immune response. Fish skin-derived ADM lacks the α-Gal antigen and poses a low risk of viral infection, making it an economical and sustainable source (39). Nevertheless, the primary constituents of fish skin-derived ADM are generally less thermally stable and more susceptible to degradation compared to human-derived ADMs (40). Bovine-derived ADM offers broader sourcing options and lower costs in comparison with human-derived alternatives. It is considered a favorable choice for repairing eyelid contractures due to its good short-term results; however, there is insufficient literature on the long-term safety and effectiveness of bovine ADM grafts available currently (41). Given the hist skin and human skin as well as its economic feasibility factor into consideration in clinical practice settings; pigs have become the primary source for xenogeneic ADM.

ADM products have been developed to mimic the extracellular matrix (ECM) of the host. Table 2 provides an overview of the currently available and widely used ADM products in the market (33, 42–44). Among them, Alloderm and Cortiva are two commonly utilized commercial ADM products (45). The natural animal dermal matrix offers abundant sources and low cost, which presents significant advantages for clinical development and holds immense application prospects.

**Table 2:** Commercial ADM products currently available in the market include the following examples.

Product	Manufacturer	Origin
Alloderm	LifeCell Corp., Bridgewater, NJ, USA	Human dermis
Cortiva	RTI Surgical, Alachua, FL, USA	Human dermis
NeoForm	Mentor, USA	Human dermis
SurgiMend	TEI bioscience USA	Bovine dermis
Permacol	Covidien Co., Ltd, USA	Porcine dermis
Strattice	LifeCell Co., Ltd, USA	Porcine dermis
CollaMend	CR Bard Davol Co., Ltd, USA	Porcine dermis
XenMatrix	CR Bard Davol Co., Ltd, USA	Porcine dermis
DermACELL	LifeNet Health Inc., Virginia Beach, VA, USA	Human dermis
Cymetra	LifeCell Corp., Bridgewater, NJ, USA	Human dermis
DermaMatrix	Musculoskeletal Transplant Foundation and Synthesis, USA	Human dermis
MatriDerm	Dr Suwelack AG, Billerbeck, Germany	Bovine dermis
Glyaderm	Euroskinbank, NL	Human dermis
Pelnac	Gunze Corp., JP	Bovine dermis
Renoskin	Perouse Plastie, FR	Bovine dermis
AlloMax	CR Bard/Davol Inc., Cranston, RI, USA	Human dermis
FlexHD	Ethicon, Inc., Somerville, NJ, USA	Human dermis
Renov	Beijing Qingyuan Weiye Bio-tissue Engineering Co., Ltd, China	Porcine dermis
Integra	Integra Life Sciences, Princeton, NJ, USA	Bovine dermis
Porcine ADM dressing	Jiangyin Benshine Biological Technology Co., Ltd, China	Porcine dermis
Allogenic ADM	Beijing Jayyalife Biological Technology Co., Ltd, China	Human dermis
Xenogeneic ADM dressing	Jiangsu Unitrump Bio-medical Technology Co., Ltd, China	Porcine dermis

## 4 Characteristics and advantages of ADM

### 4.1 Characteristics

ADM removes cellular elements that have potential immunogenicity while retaining the original extracellular matrix as a supportive framework (46). This three-dimensional structure comprises biomaterials such as collagen, elastin, and proteoglycan that serve as an ideal substrate for epithelial cell growth, fibroblast proliferation, and neovascularization post-transplantation (47). ADM can be regarded as a collagen-based scaffold with preserved three-dimensional structure obtained through physical-chemical-biological methods involving elimination of epidermal cells and other constituents from natural skin tissue. Consequently, ADM possesses slight variations in its physicochemical characteristics compared to native tissues; particularly exhibiting inferior mechanical properties along with reduced resistance against enzymatic degradation (48).

The xenogeneic ADM undergoes a process where immunogenic cells and skin appendages are removed, resulting in the presence of mainly collagen, laminin, hyaluronic acid, elastin, keratan sulfate, fibronectin, and a small amount of cell-associated proteins along with abundant type I collagen (49, 50). Being a xenogeneic protein, the telopeptide of this however, numerous experiments have demonstrated that the immunogenicity of collagen is relatively weak. The primary constituent found in ADM is collagen I which exhibits excellent histocompatibility (51). It should be noted that xenogeneic ADMs elicit a more severe inflammatory response compared to allografts possibly due to the presence of basement membrane constituents such as collagen IV and laminin in the superficial dermis.

In practical applications, ADM often requires properties similar to native tissue in order to meet the specific requirements. Therefore, for better application performance, a certain degree of cross-linking modification is advantageous in further reducing the immune response triggered by the material. Commonly used modification methods include physical and chemical approaches, with thermal modification, radiation irradiation, and drilling being the primary physical methods. Chemical methods involve crosslinking modifications using glutaraldehyde, epoxides, carbodiimide, nanomaterials, dialdehyde polysaccharides and others. Previous studies have demonstrated that cross-linking can effectively decrease immunogenicity by inducing covalent bond formation between amino acid residues in ADM and collagen molecules which can mask tissue antigenicity and reduce immune responses (52).

Chen et al. (53) employed chemical cross-linking and the synergistic binding of ADM and chitosan under freezing conditions to fabricate a composite scaffold with dual physical and chemical, thereby significantly enhancing the survival rate of autologous rat skin grafts. Feng et al. (54) synthesized glutaraldehyde-modified heparin with cross-linked active aldehyde groups, which was subsequently cross-linked with porcine ADM and chemically modified to enhance its anticoagulant performance. The results demonstrated that the modified ADM exhibited superior thermal stability and biocompatibility, particularly in terms of its enhanced anticoagulant and anti-platelet adhesion properties, leading to a reduced incidence of coagulation, thrombocytopenia, and bleeding. Improved properties can be achieved through the utilization of newly developed nanoengineered ECM scaffold technologies (55, 56). The complete elimination of immunogenicity in ADM cannot be guaranteed solely by removing cellular components alone. The rate at which residual cellular components are removed from dermis can be enhanced by increasing temperature differentials and altering freeze-thaw cycles (57).

### 4.2 Advantages

The development of ADM aims to harness the properties of native ECM and facilitate tissue regeneration in practical clinical settings. ADM exhibits properties such as toughness, elasticity, water retention, and robust mechanical force buffering (58). It can serve as a dermal scaffold, facilitating the rapid migration and infiltration of various host cells including fibroblasts, myofibroblasts, lymphocytes, macrophages, granulocytes, mast cells and more (47, 58,59). Following inflammatory cell infiltration, there increased levels of collagen and elastin production leading to the formation of new connective tissue and blood vessels (61). ADM demonstrates excellent biological strength and effectively supports fixation while reducing tension during cell proliferation (58).

Secondly, ADM harbors a multitude of signaling factors, such as vascular endothelial growth factor (VEGF), transforming growth factor β (TGF-β), basic fibroblast growth factor (BFGF), and others (62). These growth factors have demonstrated the ability to retain their biological activity post-sterilization and during extended storage, while also promoting cell adhesion, proliferation, differentiation, and tissue formation (59). Additionally, ADM exhibits low antigenicity and excellent histocompatibility, creating an advantageous environment for the growth and proliferation of seed cells (63, 64). Following implantation in the body, ADM gradually undergoes degradation and is subsequently replaced by new tissue. Compared to other biological materials, ADM provides ample space for normal cell growth, making it a suitable candidate for use as a biological injection material (65–67). ADM can be considered as an “off-the-shelf” natural biomaterial that effectively addresses the issue of donor site insufficiency within the host itself (68). In summary, ADM scaffolds possess a complex composition consisting of diverse molecules that play pivotal roles in the process of tissue regeneration.

## 5 The application of ADM in urethral repair and reconstruction for tissue engineering

Traditional techniques for urethral reconstruction involve the utilization of external genital skin, oral mucosa, and bladder mucosa for repair. However, this approach a risk of necrosis in the transplanted skin and mucosal tissue. Moreover, it fails to achieve functional restoration of the urethral epithelium (69, 70). Tissue engineering techniques offer the advantage of not necessitating large quantities of autologous tissue, particularly in cases where an increase in urethral length is required instead of autologous tissue harvesting. ADM is a three-dimensional extracellular matrix framework derived from the skin that serves as a reparative material. Although initially used in patients with severe burns, ADM has recently gained widespread application across various clinical fields including plastic surgery, breast surgery, head and neck surgery, otolaryngology, urology, oral surgery abdominal wall reconstruction gynecology and ophthalmology (71–77). ADM has gradually become a popular choice for urethral repair and reconstruction in tissue engineering. Currently scaffold-based urethral reconstruction emphasizes the use of either cell-free or cell-seeded ADMs.

### 5.1 Using cell-free ADM scaffold material

In the initial stage, Lin et al. (78) successfully achieved the reconstruction of 16 male urethral diseases by suturing ADM into a tubular structure, which included 13 complicated urethral strictures and one urethral hypospadias. The average length of urethral replacement was 4.7 cm, ranging from 2 to 10 cm. Postoperative urethrography demonstrated excellent integration of ADM with the surrounding tissue, while urethroscope examination revealed complete epithelialization of the graft urethra. No postoperative infections or rejections were observed; however, three cases experienced postoperative urethral strictures that were effectively managed through dilatation or incision. ADM is tentatively considered an ideal substitute for the urethra. On the other hand, Liu et al. (79) implanted sutured allogeneic ac dermal matrix into a tubular structure to repair 5.0 cm long urethral defects in 15 dogs. After a follow-up period of 24 weeks, no infections or anastomotic stenosis occurred, and there was no histological evidence of rejection with significant infiltration of inflammatory cells at any time point during evaluation. However, it should be noted that achieving normal structural approximation in the central area of ADM after transplantation requires an extended duration. Yang et al. (80) surgically treated five patients with anterior urethral strictures ranging from 5 to 9 cm in length by excising the affected segment and replacing it with tubular ADM. Three patients achieved successful voiding after catheter removal, while the remaining two underwent intermittent urethral dilation for a period of 2 months. All five patients demonstrated satisfactory voiding function 3 months post-operation, as confirmed by urethrography which revealed excellent continuity of the reconstructed urethra. ADM as a substitute for autologous tissue is a safe, effective, invasive, and cost-efficient method to avoid the trauma associated with harvesting autologous tissue in previous procedures. However, long-term outcomes regarding urethral stenosis remain uncertain.

Tang et al. (81) conducted a review of 49 patients with urethral strictures ranging from 1.5 to 3.8 cm in length who underwent urethroplasty with ADM and were followed up for 12 months. Cystoscopy revealed satisfactory coverage of the urethral epithelial mucosa in 11 cases. Infection occurred in two cases within the postoperative period of 2–4 weeks, while one case developed a urethral fistula at 5 months and seven cases experienced non-infective urethral strictures between 6 and 10 months after surgery. In this study, one out of two patients who developed postoperative infection had a stricture length of 3.0 cm, while the patient with a urethral fistula also presented with a stricture length close to 3.0 cm. Therefore, the use of ADM without seeded cells in an Onlay method for urethral reconstruction may have limited therapeutic efficacy for strictures longer than 3.0 cm. Additionally, the health status of the urethral bed should also be taken into consideration (82). El-Kassaby et al. (83) reported that acellular matrix would be suitable for use in surgical treatment of early strictures with an apparently healthy urethral bed and minimal spongiofibrosis. Primary ADM should not be performed in patients with preoperative infection complicated by urethral stones due to long-term stone irritation and poor local mucosal conditions, which increase the risk of infection or poor healing outcomes (81). In general, ADM represents a novel approach for material selection in urethral repair and reconstruction that offers benefits such as straightforward implementation, convenience, minimal invasiveness, and no additional sampling.

The most direct tissue engineering strategy for urethral reconstruction involves the utilization of natural or synthetic cell-free scaffolds, which are subsequently infiltrated with host cells and eventually degraded to form new tissue. The successful implantation of cell-free grafts relies on a healthy urethral bed, sufficient vascular supply, and the absence of spongiform fibrosis; otherwise, there is a risk of graft atrophy, inadequate tissue regeneration, and fibrosis. Considering that urethral stricture is characterized by ischemic cavernous fibrosis as a pathological process, it may impact the quality of the newly constructed urethra. Therefore, this simplified procedure can only be considered as an option for patients with short to moderate urethral defects. Clinical data demonstrates a high failure rate in treating urethral strictures longer than 4.0 cm.

The utilization of acellular fetal skin offers potential advantages compared to other acellular matrices. Sobhani et al. (84) employed an early gestational age fetal ADM scaffold for repairing hypospadias in a rabbit model. The decellularized fetal skin demonstrated favorable angiogenesis and re-epithelialization, resulting in reduced postoperative complications and shortened operation time, thereby establishing a solid foundation for treating complex hypospadias. The composition of the fetal dermis plays a critical role in scar-free wound healing and reducing complications associated with fetal dermal grafting (85). There is a significant disparity between the extracellular matrix of adult skin and fetal skin, primarily due to type Ⅰ collagen being the primary component. However, compared to adult skin, fetal skin exhibits higher levels of type Ⅲ collagen, type Ⅰ collagen, and cutin (86), which may explain its suitability as a “ready-made” material for tissue engineering urethra. Furthermore, recent studies have shown that utilizing ADM grafts in the second stage repair of hypospadias for ventral side elongation effectively corrects ventral curvature without increasing the risk of urethroplasty complications while also providing aesthetic benefits (87).

In order to enhance the efficacy of ADM materials, scholars have compared various preparation methods. Morgante et al. (88) conducted a study on porcine urethral repair using ADM and compared two types of acellular matrices: full-thickness porcine bladder matrix (PABM) and commercially derived cross-linked porcine dermal matrix (Permacol™). Anatomical and immunohistochemical evaluations were performed 3 months after the operation. The PABM graft showed complete fusion, while the Permacol™ graft remained palpable. Immunohistochemical analysis demonstrated a non-inflammatory remodeling response to both biomaterials. PABM implants displayed extensive infiltration of interstitial cells and neovascularization with significantly higher cell density than Permacol™. We believe that the poor growth of cells in Permacol™ may be attributed to variations in decellularization methods employed by different research groups, resulting in differential effects on cell removal, extracellular matrix composition, and subsequent tissue remodeling. It is evident that natural acellular matrices hold great promise as biomaterials for reconstructive and regenerative surgery; however, their physical and biological properties can potentially be influenced by chemical or radiation exposure. To fully realize the clinical potential of ADMs, it is crucial to conduct comprehensive evaluations of their safety and efficacy both *in vitro* and *in vivo* while also establishing standardized protocols for their utilization in clinical practice (Table 3).

**Table 3:** Experimental and human studies with cell-free ADM.

Authors	Source of matrix	Subjects	Usage	Techniques	Graft extension	Summary findings
Lin et al. (78)	Human dermis	Human	Urethral stricture (15 cases), Hypospadias (1 case)	Tubular^a^	2–10 cm	ADM is provisionally deemed to be an optimal alternative for the urethra
Liu et al. (79)	Dog dermis	Dog	Urethral defect (15 cases)	Tubular	5.0 cm	ADM may be an ideal tissue engineering material for the replacement of urethral
Yang et al. (80)	Human dermis	Human	Urethral strictures (5cases)	Tubular	5–9 cm	Using ADM as a substitute for autologous tissue is a safe, effective, minimally invasive, and cost-efficient method to avoid the trauma associated with harvesting autologous tissue in the past.
Tang et al. (81)	Human dermis	Human	Urethral strictures (49 cases)	Onlay^b^	1.5–3.8 cm	ADM represents a novel approach for the selection of materials in urethral repair and reconstruction. It offers the benefits of straightforward implementation, convenience, minimal invasiveness, and no additional sampling
Lin et al. (89)	Bovine dermis	Human	Hypospadias (35 cases)	Coverage	3.0 cm × 1.5 cm	Use of ADM may be a safe and efﬁcient covering technique to provide an additional coverage layer for proximal hypospadias repair, thereby reducing the incidence of ﬁstula formation, especially among patients who have poor-quality covering materials
Wang et al. (90)	Bovine dermis	Beagle	Urethral defect (21 animals)	Onlay	3.0 cm	ADM patches modiﬁed with CBD-VEGF demonstrated an optimized tissue repair performance in a way to increase tissue angiogenesis and maintain urethral function without inducing severe inﬂammation and scar formation
Sobhani et al. (84)	Human fetal dermis	Rabbit	Hypospadias (8 animals)	Onlay	4 × 4 mm	The application of acellular fetal skin (AFS) is a safe and feasible method that can decrease surgical time in a complex hypospadias reconstruction
Wu et al. (91)	Human dermis	Human	Hypospadias (219 cases)	Coverage	1.0–1.2 cm	It is found that HADM application can significantly reduce the incidence of urethrocutaneous fistula complications, without increasing the risk of infection and urethral stricture

^a^ Tubular graft surgery involves complete removal of the diseased segment of the urethra, followed by suturing the tubular graft in place to replace the resected segment.

^b^ Inlay grafting is performed by creating a longitudinal incision through the affected urethra, separating the urethral membrane, and using a graft to cover and repair the diseased area.

### 5.2 Using cell-seeded ADM scaffold material

Cell seeding with acellular dermal matrix is more suitable for tubular implants (92). The biological scaffolds are seeded with different types of cells, which can effectively promote cell expansion by releasing growth factors, cytokines, etc., thereby improving the mechanical properties of the grafts (93). Studies have demonstrated that in animal models, cell-inoculated matrix have been compared with uninoculated matrix, and the results are better with the former, which provides a robust empirical foundation for clinical research (94). The utilization of cell-based grafts has exhibited a remarkable 5.7-fold increase in long-term success rates compared to unseeded grafts (95). Importantly, incorporating cells within grafts has been shown to significantly reduce the incidence of stenosis, fistula formation, and infection. This can be attributed to the potential promotion of vascularization and urothelial barrier formation by cellular components, effectively mitigating local inflammation and fibrosis resulting from urine leakage. Autologous stem cells hold immense promise in urethral tissue engineering, with several scholars exploring the feasibility of combining ADM with stem cells. Fu et al. (96) employed bone marrow mesenchymal stem cells seeded on ADM, subsequently repairing rabbit urethral injuries. Histological examination using HE staining revealed that over time, the reconstructed area in the experimental group exhibited a transition from a single layer of urethral epithelial cells to multiple layers, closely resembling the structure of normal urethral tissue at 12 weeks post-operation. In contrast, the control group without the use of bone marrow mesenchymal stem cells displayed inferior urethral regeneration. Urethrography demonstrated an absence of urinary fistulas and urethral strictures or other complications in the experimental group, while such complications were observed in the control group. These findings indicate that combining bone marrow mesenchymal stem cells with ADM yields superior outcomes for repairing urethral injuries compared to using ADM alone. Therefore, ADM provides support for cell adhesion, growth, and proliferation, facilitating material exchange and signal transduction pathway formation. Both cellular-matrix interaction and tissue engineered graft-host tissue environment interaction are crucial factors for successful outcomes in urethral reconstruction. However, the mechanism by which seed cells participate in the regeneration process after binding to ADM remains unclear. Although preclinical animal studies tend to suggest that cell-bound ADM is more effective with fewer complications, these findings have not been substantiated by a limited number of clinical studies.

Currently, there is a limited number of clinical reports available on the construction of urethra using cell-loaded ADM grafts. Liu et al. (79) sutured allogeneic acellular dermal matrix into a tube to repair two patients with urethral defects measuring 12.0 cm and 12.7 cm in length, respectively. Urethral mucosa homogenate and bladder mucosa homogenate were applied on the inner surface of the ADM cavity, respectively. No anastomotic stenosis was observed. In the clinical application of long segment urethral reconstruction using ADM, the repair process relies on host cell regeneration at both ends, resulting in time-consuming procedures. However, by utilizing homogenization seeding of host urothelium and multicentric growth of epithelium based on nutrients infiltrated into the ADM, it is possible to effectively shorten the repair time. Fossum et al. (97) conducted a study in which autologous urethral epithelial cells were cultured *in vitro* and subsequently transplanted onto ADM for surgical treatment of 6 patients with severe hypospadias, aged between 14 and 44 months. All patients underwent a two-stage surgical approach. Initially, urothelial cells were obtained through bladder lavage and then seeded onto ADM. In the second operation, an ADM scaffold containing urothelial cells was implanted to construct a new urethra. During the follow-up period of 3.5–5 years, one patient experienced partial stenosis without receiving any specific treatment, one patient developed proximal anastomotic obstruction, and two patients developed urinary fistula requiring surgical correction. Urethroscopy performed on all patients revealed widening of the newly constructed urethra, while biopsy results from three patients indicated that the inner mucosa consisted of urothelial cells. The authors Bhargava et al. (82) performed surgical treatment on five patients with urethral strictures ranging from 4 to 11 cm buccal mucosa keratinocytes and fibroblasts on ADM. Buccal mucosa biopsies were obtained from each patient for cell collection. These grafts were utilized for two-stage procedures (*n* = 3) and urethroplasty (*n* = 2). Results revealed that two patients experienced complications of urethral fibrosis and constriction, resulting in complete or partial removal of the graft. Three other patients required internal fixation after a follow-up period of 33 months. The application of tissue engineering techniques for implanting expanded cells on ADM can effectively enhance urethral tissue regeneration and accelerate lesion healing, making it particularly suitable for patients with long and complex urethral strictures or defects. However, the optimal conditions for cell differentiation and maturation in cell-loaded ADM remain unclear, and there are stringent requirements for *in vitro* cell culture, especially when considering human treatment. Once a tissue-engineered urethra is successfully constructed *in vitro*, timely scheduling of the implantation procedure is crucial to prevent graft failure. Additionally, it should be noted that using cell-inoculated matrices would result in higher overall costs compared to using cell-free matrices.

Another aspect that has received limited attention in the design of tissue engineered urethral scaffolds is the prevention of urinary fistulas. Recent research indicates that the utilization of ADM derived from bovine skin as a coverage material for proximal hypospadias repair can effectively decrease the occurrence of urinary fistula formation (89). Wu et al. (91) investigated the use of ADM in hypospadias repair and demonstrated a significant reduction in urinary fistula complications without an increased risk of infection and urethral stricture. Given the detrimental effects of urine on the cellular components of tissue-engineered urethra, it is crucial for the scaffold to possess sufficient impermeability as an isolation barrier. Furthermore, appropriately modified ADM materials can effectively decrease the incidence of associated complications and other adverse reactions.

Wang et al. (90) utilized bovine ADM loaded with collagen binding vascular endothelial growth factor (CBD-VEGF) for the repair of canine urethral injury. A few months later, the group with urethral injury was compared to the groups receiving ADM implantation and CBD-VEGF modified ADM implantation, respectively. The findings revealed that while the control group experienced urethral stricture and diverticulum, one case in the ADM group developed urinary fistula. However, no associated complications or adverse reactions were observed in the CBD-VEGF modified ADM implantation group. Importantly, ADM effectively prevented both urethral stricture and diverticulum when compared to the control group, demonstrating its efficacy in averting these conditions. Notably, combining ADM with growth factors promotes functional vascular system formation, thereby maintaining extracellular matrix metabolism balance and facilitating urethra remodeling without inducing severe inflammation or scar hyperplasia (Table 4).

**Table 4:** Experimental and human studies with cell-seeded ADM.

Authors	Types of cells	Source of matrix	Subjects	Usage	Technique	Graft extension	Summary findings
Fu Jinshan et al. (96)	Bone marrow mesenchymal stem cells (BMSCs)	Rabbit dermis	Rabbit	Urethral injury (36 animals)	Tubular	2.0 cm × 1.0 cm	Overall findings indicate that the combination of BMSCs and acellular dermal matrix has better efficacy than the acellular dermal matrix alone
Liu et al. (79)	Urethral mucosa and bladder mucosa cells	Human dermis	Human	Urethral defects (2 cases)	Tubular	12.0 cm–12.7 cm	The repair time can be significantly reduced by employing the host urothelium homogenate seeding technique
Fossum et al. (97)	Urothelial cells	Human dermis	Human	Hypospadias (6 cases)	Tubular	25 cm^2^	This technique is feasible for treatment of a selected group of hypospadias where pronounced chordee and shortage of preputial and penile skin
Bhargava et al. (82)	Keratinocytes and fibroblasts	Human dermis	Human	Urethral stricture (5 cases)	Onlay	4–11 cm	This pilot study has identified the potential clinical usefulness of TEBM

## 6 Conclusions

Given the limited availability of tissue, coupled with a high incidence rate and challenges in achieving complete structural and functional restoration of the urethra, treatment outcomes for long urethral strictures remain unsatisfactory. ADM-based tissue engineering solutions have emerged as promising alternatives. ADM is derived from natural extracellular matrix (ECM), exhibiting exceptional biocompatibility along with appropriate mechanical properties and controlled non-toxic degradability. ADM creates a favorable microenvironment conducive to nurturing urethral parietal cell components while significantly enhancing the development of tissue-engineered substitutes for the urethra. However, further investigation is warranted in refining preparation technologies aiming to more accurately replicate ADM's inherent extracellular environment. Additionally, it is imperative to implement suitable modifications including integration of bioactive molecules.

The acellular allogeneic dermal matrix is highly versatile and readily available as an off-the-shelf material. It is the most commonly utilized scaffold type in clinical practice, devoid of cells. In terms of surgical approach, inlay grafting should be preferred over tubular reconstruction due to the latter's high failure rate. This is primarily attributed to the limited extent for adequate tissue regeneration on the stroma from the urethral wall boundary, which has been reported to be approximately 1.5 cm. We consider acellular allogeneic dermal matrix without cells as a valuable alternative when there is a scarcity of tissue sources, increased risk of donor site complications, and unsatisfactory outcomes in treating long urethral strictures. On the contrary, recent reports have highlighted the utilization of cell-seeded matrices in urethral reconstruction, indicating advancements in cell biology and biomaterials as scaffolds. Despite not fully replicating the intricate ECM microenvironment, animal studies on tissue-engineered urethra have successfully incorporated growth factors and cell co-culture. However, there are only three documented cases of cell-seeded scaffolds being used for human urethroplasty with a limited number of patients. Only 2 study that used a tubularized ADM as a construct has been published in the literature. In two cases where patients had urethral defects longer than 12.0 cm, repair was performed using acellular allogeneic dermal matrix seeded with homogenized urethral mucosa and bladder mucosa. Notably, no instances of postoperative urethral stricture were observed among these patients—an occurrence rarely reported in clinical practice (79). Additionally, acellular dermis seeded with keratinocytes and fibroblasts was employed to treat five patients with urethral stricture; however, all experienced recurrent strictures (92). The failure can be attributed to the progressive and incurable nature of lichen sclerosis itself. Additionally, other contributing factors to this outcome may include the preparation of acellular dermal matrix, selection of cell type for inoculation, and surgical technique employed. A study involving six children underwent hypospadias repair using urothelial cells inoculated into acellular dermis (94). In this study, one child required surgical intervention due to urethral restenosis, while two other patients underwent correction for urethral fistula. Human studies utilizing acellular allogeneic dermal matrices seeded with cells failed to replicate the promising results observed in animal models. However, we discovered that applying ADM coverage during urethroplasty significantly reduced the incidence of urinary fistula after hypospadias surgery—an aspect that has been relatively overlooked. In conclusion, cell scaffolds seem to hold promising prospects in the future. In particular, tubular scaffolds constructed from co-cultured cells may be a more appropriate orientation for TE urethral reconstruction.

The treatment of urethral stricture depends on a variety of factors, including patient age, etiology, length and location of stricture, preoperative intervention, and surgical experience. The results of clinical studies of ADM Tissue Engineering grafts remain uncertain due to the inadequacy of available trials. However, through the combination of ADM with an optimized decellularization protocol, novel nanoengineering technology, and 3D bioprinting technology, it holds great promise as a material for tissue-engineered urethral repair. The utilization of 3D bioprinting can simplify the creation of seeded tubular urethral structures, enhance patient-specific design options, and improve the efficiency of generating tissue-engineered urethra.
